# Interleukin-2 enhancer binding factor 2 negatively regulates the replication of duck hepatitis A virus type 1 by disrupting the RNA-dependent RNA polymerase activity of 3D polymerase

**DOI:** 10.1186/s13567-024-01294-x

**Published:** 2024-03-26

**Authors:** Hao An, Xiaoli Yu, Jing Li, Fuyan Shi, Yumei Liu, Ming Shu, Zihan Li, Xiaohong Li, Wanwei Li, Junhao Chen

**Affiliations:** School of Public Health, Weifang Medical University, Weifang, 261042 Shandong China

**Keywords:** Interleukin-2 enhancer binding factor 2, 3D polymerase, RNA-dependent RNA polymerase, Duck hepatitis A virus type 1, 3′—untranslated region

## Abstract

The interaction between viral components and cellular proteins plays a crucial role in viral replication. In a previous study, we showed that the 3′—untranslated region (3′—UTR) is an essential element for the replication of duck hepatitis A virus type 1 (DHAV-1). However, the underlying mechanism is still unclear. To gain a deeper understanding of this mechanism, we used an RNA pull-down and a matrix-assisted laser desorption/ionization time-of-flight mass spectrometry assay to identify new host factors that interact with the 3′—UTR. We selected interleukin-2 enhancer binding factor 2 (ILF2) for further analysis. We showed that ILF2 interacts specifically with both the 3′—UTR and the 3D polymerase (3D^pol^) of DHAV-1 through in vitro RNA pull-down and co-immunoprecipitation assays, respectively. We showed that ILF2 negatively regulates viral replication in duck embryo fibroblasts (DEFs), and that its overexpression in DEFs markedly suppresses DHAV-1 replication. Conversely, ILF2 silencing resulted in a significant increase in viral replication. In addition, the RNA-dependent RNA polymerase (RdRP) activity of 3D^pol^ facilitated viral replication by enhancing viral RNA translation efficiency, whereas ILF2 disrupted the role of RdRP in viral RNA translation efficiency to suppress DHAV-1 replication. At last, DHAV-1 replication markedly suppressed the expression of ILF2 in DEFs, duck embryo hepatocytes, and different tissues of 1 day-old ducklings. A negative correlation was observed between ILF2 expression and the viral load in primary cells and different organs of young ducklings, suggesting that ILF2 may affect the viral load both in vitro and in vivo.

## Introduction

Duck viral hepatitis (DVH) is an acute, rapidly spreading, fatal disease of young ducklings characterized primarily by liver necrosis, hemorrhage, and high mortality. DVH was first described in Long Island in 1949 [1], and the causative agent of this fatal disease was later divided into duck hepatitis virus type 1 (DHV-1), type 2 (DHV-2), and type 3 (DHV-3) [2, 3]. DHV-1 was initially classified as an *enterovirus* based on the virion’s observed morphology and physicochemical properties [4]. It was then classified as a member of the new genus *Avihepatovirus* in the family *Picornaviridae* and renamed duck hepatitis A virus (DHAV) in Virus Taxonomy: the Ninth Report of the International Committee on Taxonomy of Viruses (ICTV) [5]. Based on phylogenetic analyses and neutralization tests, DHAV was then genetically divided into three distinct types: DHAV-1 (the original serotype 1) [6], DHAV-2 (a serotype isolated in Taiwan) [7], and DHAV-3 (a serotype isolated in South Korea and China) [8].

The complete genome of DHV-1 consists of approximately 7000 nucleotides in a positive, single-stranded, uncapped RNA with a 3′-poly(A) tail. The genome structure of DHAV-1 is similar to that of other members of the *Picornaviridae* family, i.e., with an open reading frame flanked by 5′- and 3′-untranslated regions (UTRs). The open reading frame encodes three structural proteins (VP0, VP1, and VP3) and nine nonstructural proteins (2A1, 2A2, 2A3, 2B, 2C, 3A, 3B, 3C, and 3D) [6]. The 3′-UTR plays an important role in regulating viral replication and internal ribosome entry site-mediated translation efficiency of DHAV-1 [9]. The 3D polymerase (3D^pol^), which has RNA-dependent RNA polymerase (RdRP) activity, is also a crucial determinant of DHAV-1 viral replication [10]. In addition, the 3D^pol^ specifically interacts with the 3′-UTR of positive viral strands to synthesize negative strands, which are then used to produce numerous positive-stranded RNAs [11, 12].

The cellular protein interleukin-2 enhancer binding factor 2 (ILF2), also known as nuclear factor 45 (NF45) in humans and mice, is a constitutively expressed protein that interacts with chromatin. ILF2 is a nuclear factor of activated T cells that regulates the transcription of the *IL2* gene in the nucleus during T cell activation by forming a stable heterodimer with interleukin enhancer binding factor 3 (ILF3, also known as NF90) [13]. The ILF2/ILF3 complex is a crucial regulatory factor involved in apoptosis [14], DNA repair [15], gene transcription [16], microRNA processing [17, 18], mRNA translation [19], and host defense [20]. In addition, the ILF2/ILF3 heterodimer was reported to regulate the replication process of numerous viruses, such as hepatitis C virus [21], infectious bursal disease virus (IBDV) [22], poliovirus [23], influenza virus [24], dengue virus [25], human immunodeficiency virus type 1 [26], human T-cell leukemia virus [27], porcine reproductive and respiratory syndrome virus (PRRSV) [28], and Japanese encephalitis virus [29]. Interestingly, ILF2 functions as both a negative regulator [28, 29] and a positive regulator [26] in the viral replication process. The full-length cDNA of duck ILF2 (XM_038167928.1) consisted of a 1173 bp open reading frame encoding a protein composed of 391 amino acid residues, with a predicted molecular weight of 42.9 kDa. Comparing the duck ILF2 nucleotide to human ILF2 (NM_004515.4) and mouse ILF2 (NM_026374.3) revealed identities of 76.1% and 82.3%, respectively. In terms of amino acid sequence, duck ILF2 shared 96.4% similarity with both human and mouse ILF2. However, to the best of our knowledge, its specific role during the DHAV-1 infection has yet to be elucidated.

Viral replication requires a series of protein–RNA and protein–protein interactions. Currently, little is known about virus–host interactions during the replication of DHAV-1. In this study, we identified ILF2 as a regulator of the viral tissue tropism of DHAV-1 by negatively regulating viral replication through disrupting the RdRP-mediated promotion of viral RNA translation efficiency. Elucidation of this regulatory mechanism provides new insights into the potential role of the cellular component ILF2 in regulating viral replication by interacting with viral genome RNA and viral nonstructural proteins.

## Materials and methods

### Plasmid construction

Total RNA was extracted from 1 g of kidney tissue of 1-day-old ducklings using an E.Z.N.A.™ Total RNA Kit (Omega Bio-Tek, Norcross, GA, USA) and then used for reverse transcription using a RevertAid™ First Strand cDNA Synthesis Kit (Fermentas, Burlington, Canada). The generated cDNA of ILF2 was then used for PCR amplification using the corresponding primers (Table 1). The PCR products obtained were inserted into a pcDNA3.1/V5-His B vector, a pET-32a( +) vector, and a pCMV-myc vector (Clontech, Palo Alto, CA, USA) to generate the recombinant plasmids pCDNA3.1-ILF2, pET32-ILF2, and pCMV-myc-ILF2, respectively, after digestion with the appropriate restriction enzymes. The viral RNA of DHAV-1 strain WF1107 (GenBank no. MW462237) was used for PCR amplification to generate 3D and VP0 gene using the corresponding primers listed in Table 1. The *3D* gene was inserted into the pCMV-HA vector (Clontech) or pBudCE 4.1 vector to generate pCMV-HA-3D or pBudCE-3D after digestion with the appropriate restriction enzymes. The *3D* and *ILF2* genes were inserted into the pBudCE 4.1 vector to generate the recombinant plasmid pBudCE-3D-ILF2. The *VP0* gene was inserted into the pET-32a( +) vector to express the VP0-His fusion protein, which was then used to produce antisera against DHAV-1. The methods for purification of the His-tagged proteins and generation of mouse polyclonal antisera are described below. All recombinant plasmids were sequenced by Sangon Biotech Co., Ltd., Shanghai, China. All primers used in this study are listed in Table 1.

**Table 1:** **Primers used in this research**.

Primers	Sequence (5′—3)^a^	Purpose	Genebank Access No.^b^
Primers for reverse transcription			
RT-ILF2-R	CTCCTGAGTCTCCATGCTCTCCTCTT	RT-PCR for ILF2	XM_038167928
Primers for plasmids construction			
ILF2-EcoRI-F	ccgGAATTCGGATGGCTTTCCCGCGGGTTA	pCMV-myc-ILF2	XM_038167928
ILF2-XhoI-R	ccgCTCGAGTCACTCCTGAGTCTCCATGCTCT		
ILF2-HindIII-F	cccAAGCTTGCCACCATGGCTTTCCCGCGGGTTA	pCDNA3.1-ILF2	XM_038167928
ILF2-BamHI-R	cgcGGATCCTCACTCCTGAGTCTCCATGCTCT	PBudCE-3D-ILF2	
ILF2-BamHI-F	cgcGGATCCATGGCTTTCCCGCGGGTTA	pET32-ILF2	XM_038167928
ILF2-HindIII-R	cccAAGCTTTCACTCCTGAGTCTCCATGCTCT		
3D-BglII-F	ggaAGATCTGGGAAAGTAGTAAGCAAGCAATA	pCMV-HA-3D	MW462237
3D-NotI-R	aagGCGGCCGCTTAGATCATCATGCAAGC		
3D-NotI-F 3D-BglII-R	aagGCGGCCGCGGGAAAGTAGTAAGCAAGCAATA ggaAGATCTTTAGATCATCATGCAAGC	PBudCE-3D	MW462237
VP0-BamHI-F	cgcGGATCCATGGATACTCTTACCAAAAAC	pET32-vp0	MW462237
VP0-XhoI-R	ccgCTCGAGCTGATTGTCAAATGGTCGGGGACAC		
3UTR-F	ACTGTTGGTCCGCAGGTACCATAAA	3′ UTR of DHAV-1	MW462237
3UTR-R	AGGTAGGGTAGGGAATAGTAAA		
3UTR-reverse-F	AGGTAGGGTAGGGAATAGTAAAG	Reverse sequence of 3′ UTR of DHAV-1	MW462237
3UTR-reverse-R	ACTGTTGGTCCGCAGGTACCATAAACC		
Primers for qRT-PCR			
ILF2-qRTPCR-F	GCAGACGCTTGTCCGCATCC	qRT-PCR for ILF2	XM_038167928
ILF2-qRTPCR-R	GGTTCCTCCTGGTTCTCCTCTTCC		
DHAV-qRT-PCR-F	AAACACCCACTGGCTTTGGA	qRT-PCR for DHAV-1	MW462237
DHAV-qRT-PCR-R	GGTCCTCACGGAAAGTGGAG		
GAPDH-F	ATGTTCzGTGATGGGTGTGAA	qRT-PCR for GAPDH	XM_061080759.1
GAPDH-R	CTGTCTTCGTGTGTGGCTGT		
Primers for RNA interference			
siILF2 (sence)	UCAAUUUGCACCUCGAAAGUU	RNAi against ILF2	XM_038167928
siILF2 (antisence)	GGUAGCAGAACGUCCCACAGC		
NC siRNA (sence)	UUCUCCGAACGUGUCACUUtt	Negative control siRNA	XM_038167928
NC siRNA (antisence)	ACGUGACACGUUCGGAGAAtt		

^a^Lowercase letters represent protective bases; Restriction sites are underlined.

^b^The primers used in this research were designed based on the relevant genes.

### Cells and viruses

The duck embryo hepatocytes (DEH) were prepared using 14-to-16-day-old duck embryos. The liver tissue was generated from duck embryos and then washed three times with D-Hank’s (H1040, Solarbio, Beijing, China). The liver tissue was minced with scissors and then washed three times with D-Hank’s, followed by digestion with a solution of 0.2% trypsin at 37 °C for 4 to 6 min. The tissue was then rinsed three times with D-Hank’s to generate DEH cells. Human embryonic kidney 293 T (HEK 293 T) cells (ATCC CRL-11268), DEH and duck embryo fibroblasts (DEFs, ATCC^®^ CCL-141) were cultured at 37 °C in 5% CO_2_ in Dulbecco’s modified Eagle medium (DMEM; Gibco, Carlsbad, CA, USA) supplemented with 10% fetal bovine serum (FBS; Gibco), 100 U/mL penicillin, and 100 µg/mL of streptomycin sulfate. The virulent DHAV-1 strain WF1107 (GenBank no. MW462237) was obtained from an outbreak of severe DVH in Weifang city, Shandong Province, China.

### Purification of his-tagged fusion proteins

The collected HEK 293 T cells or *Escherichia coli* Rosetta (DE3) cells that expressing His-tagged proteins were collected and sonicated on ice. The insoluble fraction and cell debris were removed via centrifugation at 12 000 × *g* at 4 °C for 1 min. The supernatant was collected and used for ILF2-His purification via gravity chromatography using a Ni^2+^ affinity chromatography His-bind resin (Novagen, Madison, WI, USA) according to the manufacturer’s instructions. In brief, the supernatant was placed in a gravity flow column packed with 3 mL of a Ni^2+^-NTA resin slurry (Novagen), and the His-tagged proteins were eluted with 50 mM NaH_2_PO_4_, 300 mM NaCl, and 300 mM imidazole (pH 8.0). The purified ILF2-His (44 kDa, 1.49 mg/mL) and VP0-His (29 kDa, 2.37 mg/mL) were used to produce polyclonal antisera.

### Antibodies

The monoclonal antibodies anti-His (ab154063) and horseradish peroxidase (HRP)-conjugated goat anti-mouse antibodies (ab6789) were purchased from Abcam (Cambridge, MA, USA). The rabbit HA tag polyclonal antibody (Cat No: 51064-2-AP), mouse MYC tag monoclonal antibody (Cat No: 60003), and HRP-conjugated affinipure goat anti-rabbit IgG(H + L) (Cat No: SA00001-2) were purchased from Proteintech Group Inc (Wuhan, China). The anti-β-Actin (4970S) monoclonal antibodies were purchased from Cell Signaling Technology (Beverly, MA, USA). The anti-GAPDH monoclonal antibody (Cat No. 60004-1-Ig) was purchased from Proteintech Group Inc. The FITC-labeled goat anti-rabbit IgG (H + L) (A0562, Beyotime Biotech, Beijing, China) and Cy3-labeled goat anti-mouse IgG (H + L) (A0521, Beyotime Biotech) were used in the laser scanning confocal microscopy assay. The polyclonal antisera against ILF2 or DHAV-1 were produced using Balb/c mice. In brief, ILF2-His and VP0-His fusion proteins were expressed in *Escherichia coli* Rosetta (DE3) cells (Transgen Biotech, Beijing, China) after induction with 1 mM IPTG at 37 °C for 8 h and then used to produce the polyclonal antibodies. The six-week-old Balb/c mice were separately primed subcutaneously with 100 mg of purified ILF2-His or VP0-His fusion proteins emulsified with an equal volume of Freund’s complete adjuvant (Sigma-Aldrich, St. Louis, MO, USA). Two booster immunizations with 100 mg of the fusion protein in Freund’s incomplete adjuvant were administered 2 weeks apart. Finally, the 100 mg of purified fusion protein were administered without adjuvant, and the polyclonal antisera against duck ILF2 (named anti-ILF2) or VP0 of DHAV-1 (named anti-DHAV-1 antisera) were collected 72 h later.

### In vitro RNA pull-down assay

The in vitro RNA pull-down assay was performed as described previously [30]. In brief, the Pierce magnetic RNA–protein pull-down kit (Thermo Fisher Scientific, Waltham, MA, USA) was used to perform the in vitro RNA pull-down assay. The fragment of DHAV-1 3′-UTR or its reverse sequence were amplified using the corresponding primers and then were purified using a gel purification kit (D1200, Solarbio, Beijing, China). The generated PCR fragments were then used to produce the in vitro transcribed RNA samples of the DHAV-1 3′-UTR or its reverse sequence using a T7 RiboMAX Express large-scale RNA production system (Promega, Madison, WI, USA). The RNA samples were then labeled with biotin using a Pierce RNA 3′-end dethiobiotinylation kit (Thermo Fisher Scientific) according to the manufacturer’s instructions and combined with magnetic beads. The magnetic beads-RNA-biotin mixture was then incubated with 50 mg of purified ILF2-His fusion protein, and the isolated proteins were subjected to Western blot analysis using anti-His monoclonal antibody (1:3000).

### SDS-PAGE and western blot

For SDS-PAGE analysis, the separated proteins were mixed with a 5 × SDS-PAGE sample buffer (Beyotime Biotech), and the mixture was boiled for 5 min. The samples were then subjected to electrophoresis in 10% acrylamide gels (Beyotime Biotech). For Western blot analysis, protein bands were electroblotted onto a polyvinylidene fluoride (PVDF) membrane (Thermo Fisher Scientific) using an MT transfer buffer (25 mM Tris, 0.19 M glycine, 20% methanol pH 8.0). The PVDF membrane was blocked with 5% non-fat milk in TBST (500 mL NaCl, 0.05% Tween 20, 10 mM TRIS–HCl, pH 7.5) for 1 h, and incubated with primary antibodies at 4 °C for 8 h. The membrane was then washed five times with TBST and incubated with the corresponding secondary antibodies at 25 °C for 2 h. After incubation, the PVDF membrane was washed five times with TBST, incubated with a mixture of hydrogen peroxide and 3,3′-diaminobenzidine tetrahydrochloride (Sigma-Aldrich), and visualized using an enhanced chemiluminescence system (Bio-Rad, Hercules, CA, USA). Images were captured by the BIO-RAD ChemiDoc^™^ Imaging System (Bio-Rad, Redmond, WA, USA). The band density was quantified using Image J Software (version 1.8.0, National Institutes of Health (NIH), Bethesda, MD, USA) with normalization to the β-actin or GAPDH signal.

### Laser scanning confocal microscopy

To measure the co-localization of 3D and ILF2, the DEFs in glass bottom cell culture dish (MatTek Corporation, MA, USA) were co-transfected with pCMV-myc-ILF2 and pCMV-HA-3D recombinant plasmids. At 12 and 24 h post-transfection (hpt), DEFs were washed with PBS five times and treated with Triton X-100 (ST797, Beyotime Biotech) for five min at 25 °C. The DEFs were then incubated with primary antibodies (anti-HA or anti-MYC, 1:3000) at 37 °C for 2 h, and then were incubated with the corresponding secondary antibodies (FITC-labeled Goat Anti-Rabbit IgG (H + L) and Cy3-labeled Goat Anti-Mouse IgG (H + L), 1:3000) at 37 °C for 2 h. After incubation, the DEFs were stained with 4′,6-diamidino-2-phenylindole (DAPI) for 10 min. The DEFs were washed with PBS five times and then imaged using a laser confocal microscope (Leica AF6000). The FITC signal was measured at 480 nm excitation and 520 nm emission wavelengths, while the cy3 signal was measured at 550 nm excitation and 570 nm emission wavelengths.

### CO-IP assay

The co-immunoprecipitation (CO-IP) assay was performed using a Pierce™ Classic Magnetic IP/Co-IP Kit (Thermo Fisher Scientific) according to the manufacturer’s instructions. To determine whether the 3D^pol^ of DHAV-1 specifically interacts with ILF2, equal copies of three groups of plasmids (pCMV-myc-ILF2 and pCMV-HA-3D, pCMV-myc-ILF2 and pCMV-HA vector, and pCMV-myc vector and pCMV-HA-3D) were separately co-transfected into HEK 293 T cells using Lipofectamine 2000 (Invitrogen, Carlsbad, CA, USA). At 48 hpt, the cells were gently washed once with PBS, and ice-cold IP Lysis/Wash Buffer mixed with a protease inhibitor was added to the pellet. The lysate was incubated on ice for 5 min and then centrifuged at approximately 13 000 × *g* for 10 min, and the supernatant was collected. The samples that used for measuring the expression level of ILF2-myc and 3D-HA in the three co-transfection groups were named “input group”, the supernatant was used for Western blot analysis using anti-MYC (1:3000) and anti-HA mAbs (1:3000). Samples that used for CO-IP assay were named “IH group” and were precleared via treatment with protein A/G-Sepharose beads (Pierce Biotechnology, Waltham, MA, USA) at 4 °C for 2 h. Then, anti-HA mAbs (1:3000) and protein A/G-Sepharose beads were added to the supernatant to capture the cellular proteins. The isolated proteins from CO-IP assay were then analyzed through Western blot using anti-MYC (1:3000).

To determine whether 3D^pol^ interacts with endogenous ILF2 in DEFs, 4 μg of the recombinant plasmid pCMV-HA-3D was transfected into DEFs. At 60 hpt, the cells were harvested, and a CO-IP assay with anti-HA mAbs (1:3000) was performed, followed by Western blot analysis using anti-ILF2 (1:100).

### Luciferase activity measurement

The recombinant plasmid pDHAV-3′UTR-A_25_ containing the T7 promoter, the 5′—UTR of DHAV-1, the Firefly luciferase gene, and the 3′—UTR and poly(A)_25_ tail of DHAV-1 was constructed [30]. The recombinant plasmid pDHAV-3′UTR-A_25_ was digested with restriction enzyme XhoI (TaKaRa, Dalian, China) and gel purified, and then used for in vitro transcription using the T7 RiboMAX Express large-scale RNA production system (TaKaRa). The generated RNA was then transfected into DEFs according to manufacturer instruction. To measure luciferase activity, the DEFs were washed three times with PBS and then treated with 100 μL of passive lysis buffer (Promega) for 5 min at 25 °C. The cell lysates were then collected and centrifuged at 13 000 × *g* for 5 min at 4 °C, the pellet was discarded, and the supernatant was then used to measure luciferase activity using a dual luciferase assay system (Promega) and a Berthold luminometer (GloMax 20/20; Promega).

### RNAi

DEFs (approximately 5 × 10^5^ cells in DMEM without FBS and antibiotics) were transfected with 1.25 µL siRNA against ILF2 (20 µM) using ribo*FECT*^™^ CP reagent (RIBOBIO, Guangzhou, China) according to the manufacturer’s recommendations. The DEFs in the control group were transfected with the same amount of negative control siRNA (Table 1). The siRNA against ILF2 and negative control siRNA were designed based on Anas platyrhynchos ILF2 mRNA sequence (XM_038167928). Cell lysates from both groups were collected 24 to 72 hpt and then lysed in RIPA buffer (50 mM Tris–HCl at pH 7.5, 150 mM NaCl, 1% IPEGAL, 0.5% sodium deoxycholate, protease inhibitors) on ice for 15 min followed by centrifugation at 10 000 × *g* at 4 °C for 10 min. Protein concentration was then determined using a BCA assay (Thermo Fisher Scientific), and ILF2 expression levels in both groups were then measured through Western blot using anti-ILF2 (1:100).

### Overexpression of ILF2

To upregulate the expression of ILF2, DEFs were seeded into 6-well plates, cultured until they reached 80% confluency, and transfected with Lipofectamine 2000 (Invitrogen). In brief, 4 μg of recombinant plasmids pCDNA3.1-ILF2 or 8 μL of Lipofectamine 2000 (Invitrogen) were diluted separately in 250 μL of Opti-MEM media (Thermo Fisher Scientific), and then mixed at a final volume of 500 μL after incubation for 5 min at 25 °C. The mixture was then incubated at 25 °C for 25 min and added to six-well plates. At 48 hpt, the expression level of ILF2 in both groups were measured by Western blot using anti-ILF2 (1:100).

### qRT-PCR

qRT-PCR was performed as described previously [31]. Using the primers DHAV-qRT-PCR-F/R (Table 1), a SYBR Green real-time RT-PCR assay was performed for the quantitative detection of DHAV-1 replication. Viral RNA copies were calculated according to the formula *y* =  − 3.2178*x* + 38.268, where *x* represents a standard for viral copies, and *y* represents a standard for values from one-step real-time PCR. The transcription level of ILF2 was detected using a SYBR Green real-time method. Total RNA was extracted using an E.Z.N.A.TM Total RNA Kit (Omega Bio-Tek) according to the manufacturer’s instructions. Subsequently, 1.0 µg of total RNA was used for RNA transcription using a RevertAid™ First Strand cDNA Synthesis Kit (Fermentas) according to the manufacturer’s instructions. The reverse transcription product (1.0 µM) was then used for qRT-PCR measurement using ILF2-qRTPCR-F and ILF2-qRTPCR-R primers (Table 1). The GAPDH levels were measured using GAPDH-F/R (Table 1). The PCR conditions were as follows: one cycle at 95 °C for 30 s, 40 cycles of denaturation at 95 °C for 5 s and extension at 60 °C for 34 s, followed by a dissociation curve analysis step.

### Experimental animals and sample collection

A total of twelve 1-day-old cherry valley ducklings were purchased from a local commercial poultry farm (Weifang, Shandong, China). A total of ten six-week-old Balb/c mice were purchased from a local experimental animal company (Jinan, Shandong, China). The treatment procedures of ducklings and mice were approved by the Experimental Animal Ethics Committee of Weifang Medical University (approval no. 2020SDL043) and performed in accordance with the guidelines of the Ethics Committee for Laboratory Animal Welfare of Weifang Medical University. The experimental animals were provided a basal diet and water ad libitum and managed under the recommended humidity and temperature.

To measure the impact of DHAV-1 infection on ILF2 expression, the ducklings were divided into two groups: the experimental group was injected intramuscularly with approximately 10^4^ copies of DHAV-1 viral particles, while the control group was injected intramuscularly with the same volume of PBS. The ducklings in control group or DHAV-1-infected group were housed separately. The ducklings were euthanized by carbon dioxide asphyxiation at 48 h post-infection (hpi), 1 g of the liver, kidney, heart, spleen, and bursa of Fabricius (BF) tissue were collected and used for subsequent qRT-PCR measurement. To amplify the cDNA sequence of ILF2, 1 g of the kidney tissue was collected and used for total RNA extraction and the subsequent PCR amplification. The immunized mice, which were used to produce antisera, were euthanized by carbon dioxide asphyxiation, followed by cervical dislocation. The blood samples were collected from the eyeballs, stored at 25 °C for 2 h, and then were centrifuged at 5000* g* rpm/min to produce antisera.

### Statistical analysis

All experiments were performed at least three times with at least three biological replicates. Statistical significance was determined using SPSS software (version 20.0, SPSS Inc., Chicago, IL, USA) evaluated using one-way ANOVA (three or more groups of data) and independent-sample t test (two groups of data). *P < 0.05, **P < 0.01, ***P < 0.001.

## Results

### ILF2 Interacts with the 3′-UTR of DHAV-1

**Figure 1:**
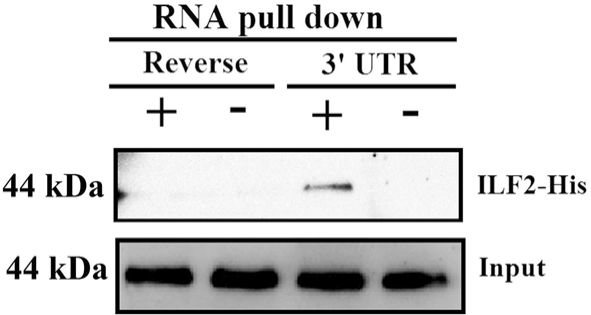
**ILF2 interacts with the 3′-UTR of DHAV-1**. Western blot analysis of the isolated products from the in vitro RNA pull-down assay. The anti-His (1:3000) and HRP-conjugated affinipure goat anti-rabbit IgG(H + L) (1:3000) were used in this assay.

To search for novel host factors that interact with the 3′-UTR of DHAV-1, an RNA pull-down assay was performed using the biotin-labeled RNA probe of the 3'-UTR. The isolated products were analyzed using matrix-assisted laser desorption/ionization time-of-flight mass spectrometry (MALDI-TOF–MS). The MALDI-TOF–MS results indicated numerous cellular proteins that might interact with 3'-UTR of DHAV-1, and ILF2 was chosen for further analysis due to its high index [30]. Next, an in vitro RNA pull-down assay was performed using the biotin-labeled RNA probe of the 3′-UTR and the purified ILF2-His fusion protein to investigate whether ILF2 specifically interacted with the 3′-UTR of DHAV-1, as described in a previous study [30]. The biotin-labeled RNA probe of the reverse sequence of the 3'-UTR and purified ILF2-His fusion protein were used as the negative control. Western blot analysis of the isolated products showed that ILF2 was immunoprecipitated in the group with the 3′-UTR probe but not in the group with the reverse 3′-UTR probe (Figure 1), indicating that ILF2 directly interacted with the 3′-UTR of DHAV-1, further confirming the results of the MALDI-TOF–MS.

### ILF2 specifically interacts with the 3D.^pol^ of DHAV-1

**Figure 2:**
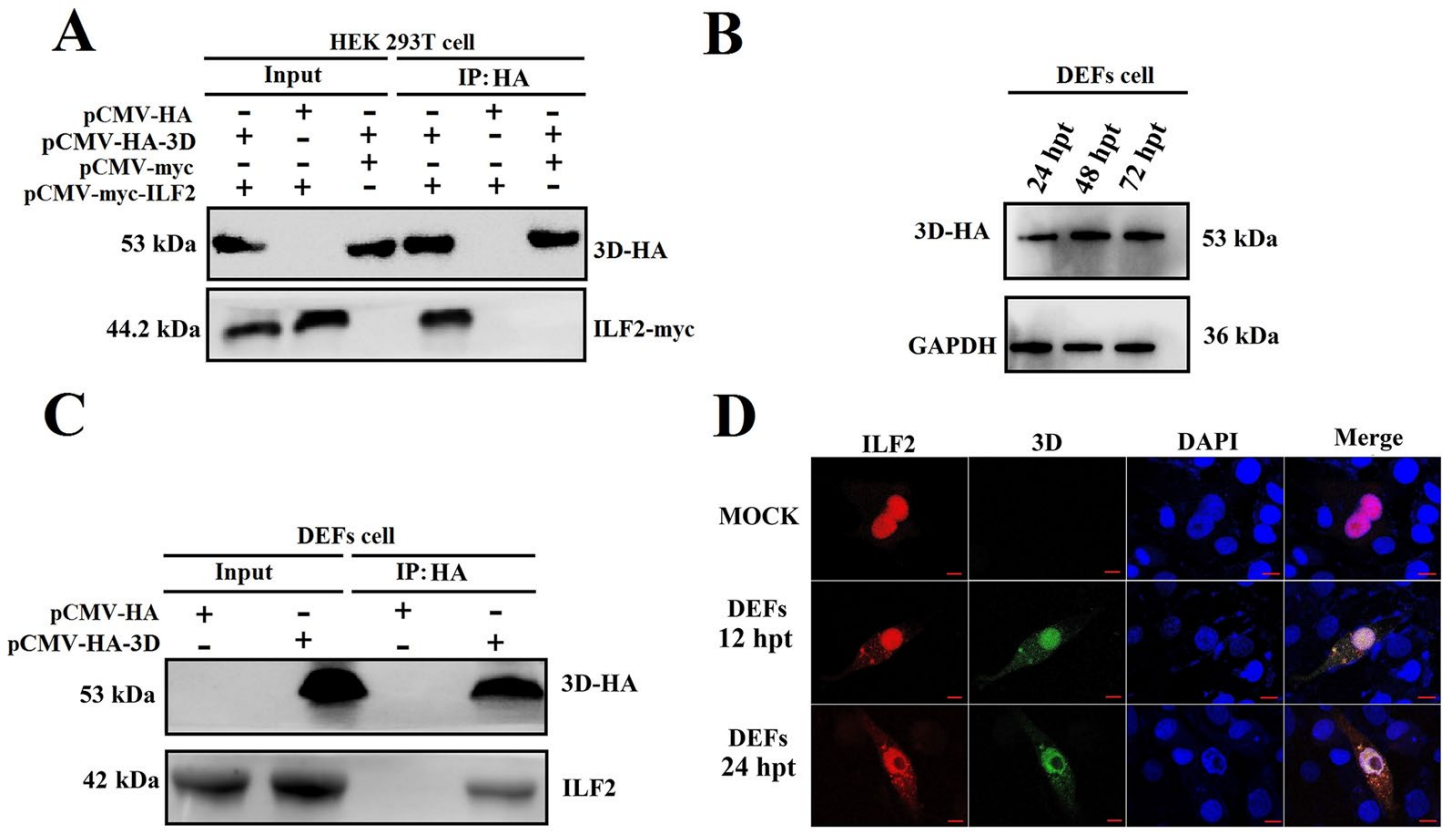
**ILF2 interacts with 3D**^**pol**^** both in vitro and in vivo**. **A** The cells in the HA group were collected for CO-IP assay using anti-HA, and the isolated proteins from the CO-IP assay were then detected by Western blot assay using anti-MYC and anti-HA were used in this assay (1:3000). The cells in the input group were lysed and subjected to Western blot analysis using anti-MYC and anti-HA. **B** The DEFs cells were transfected with 4 μg of recombinant plasmids pCMV-HA-3D, the expression level of 3D-HA at 24, 48, and 72 hpt were measured by Western blot using anti-HA mAb (1:3000). GAPDH was detected using anti-GAPDH (1:3000). **C** The cells in the HA group were collected for CO-IP assay using anti-HA mAb, and the isolated proteins from the CO-IP assay were then detected by Western blot assay using anti-ILF2 (1:100) and anti-HA (1:3000). The cells in the input group were lysed and subjected to Western blot analysis using anti-HA mAb (1:3000) and anti-ILF2 (1:100). **D** The laser confocal microscopy result of the co-localization result of 3D and ILF2 in DEFs at 12 and 24 hpt. The anti-ILF2 (1:50) were used to detect the cellular localization of endogenous ILF2 (red) in Mock DEFs (non-transfected DEFs) and DEFs transfecting with pCMV-HA-3D, while the anti-HA (1:3000) were used to measure the cellular localization of 3D-HA (green). The Mock group indicated blank DEFs, the DAPI channel indicated staining of DEFs nucleus, the Merge images represent single stack of 3D-HA (green), endogenous ILF2 (red), and DAPI (blue). Bar represents 10 μm.

A previous study showed that ILF2 interacted with other RNA binding proteins through binding to the RNA [26]. Therefore, we sought to investigate whether ILF2 interacts with 3D^pol^ using a CO-IP assay. Equal copies of three groups of plasmids (pCMV-myc-ILF2 and pCMV-HA-3D, pCMV-myc-ILF2 and pCMV-HA vector, and pCMV-myc vector and pCMV-HA-3D) were separately co-transfected into HEK 293 T cells, and ILF2-myc and 3D-HA were highly expressed in input group. Next, the CO-IP assay was conducted using anti-HA mAb, and ILF2 was immunoprecipitated in the pCMV-myc-ILF2 and pCMV-HA-3D co-transfection groups (Figure 2A), indicating that ILF2 interacted with the 3D protein in vitro. In addition, we investigated whether the 3D^pol^ of DHAV-1 interacts with endogenous ILF2. The DEFs were transfected with pCMV-HA-3D to express 3D-HA fusion protein (Figure 2B), the DEFs in control group were transfected with pCMV-HA vector. Next, the DEFs in both groups were collected at 60 hpt to perform an IP assay, followed by Western blot analysis. The results showed that endogenous ILF2 was immunoprecipitated in the 3D-expressing group but not in the control group, indicating that 3D^pol^ specifically interacted with endogenous ILF2 (Figure 2C).

Moreover, the co-localization of 3D and ILF2 was measured by transfecting the recombinant plasmids pCMV-myc-ILF2 and pCMV-HA-3D into DEFs. At 12 and 24 hpt, the co-immunolabeling results revealed that ILF2 was co-localized with 3D (Figure 2D), rendering a physical basis for their interaction.

### 3D^pol^ facilitates DHAV-1 replication by enhancing viral RNA translation efficiency

**Figure 3.**
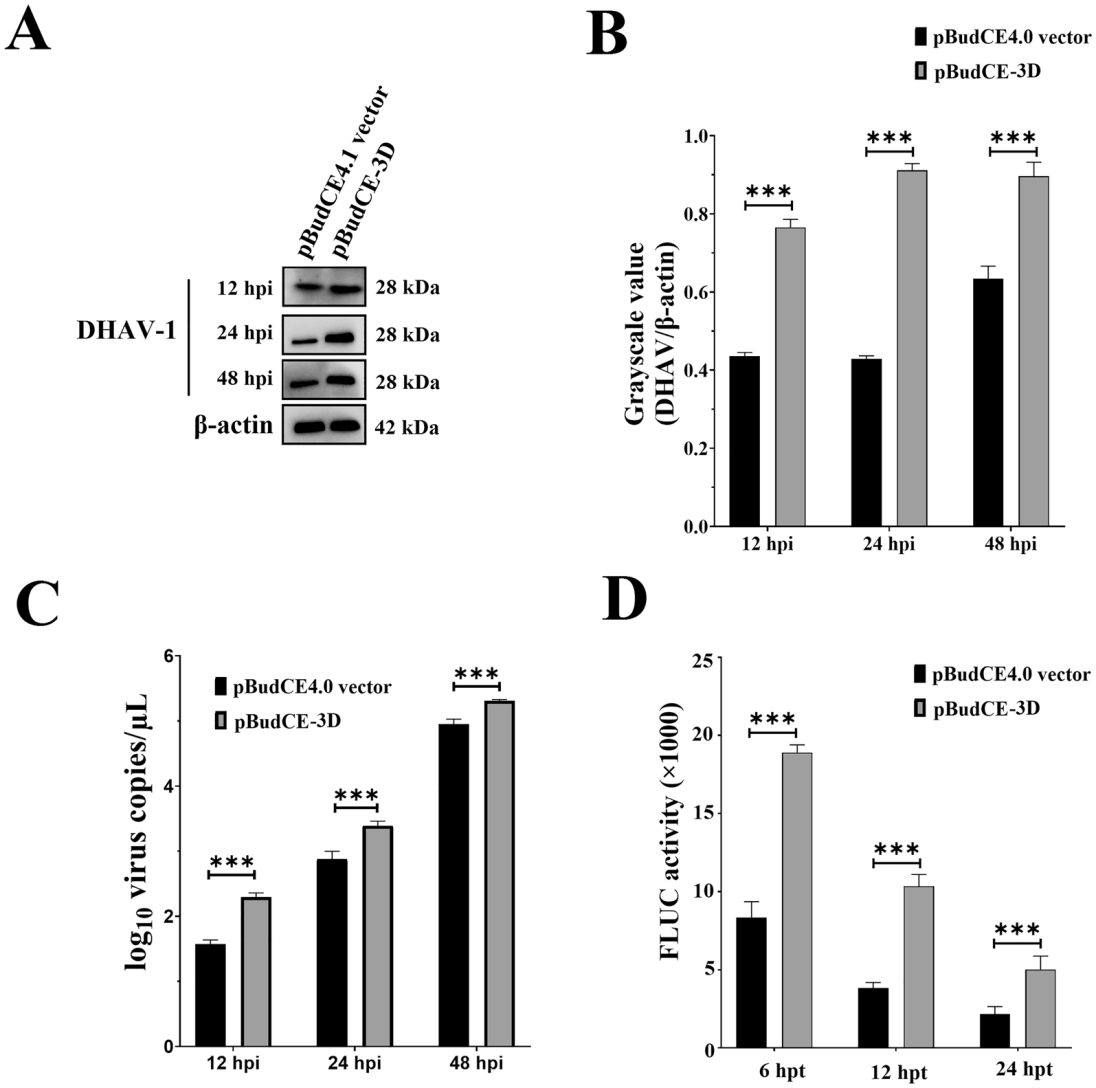
**3D**^**pol**^** facilitates DHAV-1 replication by enhancing viral RNA translation efficiency.** **A** DEFs cells were transfected with 4 μg of pBudCE-3D or pBudCE4.1 vector, and then were inoculated with 10^4^ copies of DHAV-1 at 48 hpt. The viral replication levels in both group were analyzed by Western blot using anti-DHAV-1 antiserum (1:100). **B** Viral protein levels in both groups were measured using ImageJ software, and the grayscale values corresponding to the bands of each protein between groups were conducted. The y-axis represents the ratio of the grayscale value of the viral protein to that of β-actin, whereas the x-axis represents the time, in hours post-infection. **C** qRT-PCR results indicating the number of viral copies in DEFs transfected with the pBudCE-3D or pBudCE4.1 vector. **D** Comparison of FLUC activities in DEFs transfected with the pBudCE-3D or pBudCE4.1 vector. *P < 0.05, ** P < 0.01, *** P < 0.001. Bars show the mean ± SD of three independent experiments (*n* = 3).

As demonstrated previously, 3D^pol^ exhibited RdRP activity through interacting with the 3′-UTR of DHAV-1 [10]; however, the effects of RdRP activity on DHAV-1 viral replication was still unclear. To identify the specific role of 3D^pol^ during DHAV-1 replication, DEFs were transfected with the pBudCE-3D or pBudCE4.1 vector, respectively. At 48 hpt, the DEFs in both groups were inoculated with approximately 10^4^ copies of DHAV-1, and the growth characteristics were then measured at 12, 24, and 48 hpi through Western blotting and qRT-PCR. The overexpression of 3D^pol^ prominently increased the viral replication level at all detection points (Figures 3A–C).

Next, we investigated whether 3D^pol^ could enhance viral replication by facilitating viral RNA translation. The DEFs were transfected with the recombinant plasmids pBudCE-3D or pBudCE4.1 vector. At 48 hpt, the translation reporter system 5′-UTR_FLUC_3′-UTR was transfected into both groups, and the firefly luciferase (FLUC) activity was measured at different hours post-transfection. The FLUC activity in DEFs transfected with pCMV-HA-3D was highly higher compared to that in the control group at all detection points (Figure 3D), indicating that 3D^pol^ enhanced DHAV-1 replication by increasing viral RNA translation efficiency.

### ILF2 negatively regulates the viral replication of DHAV-1

**Figure 4:**
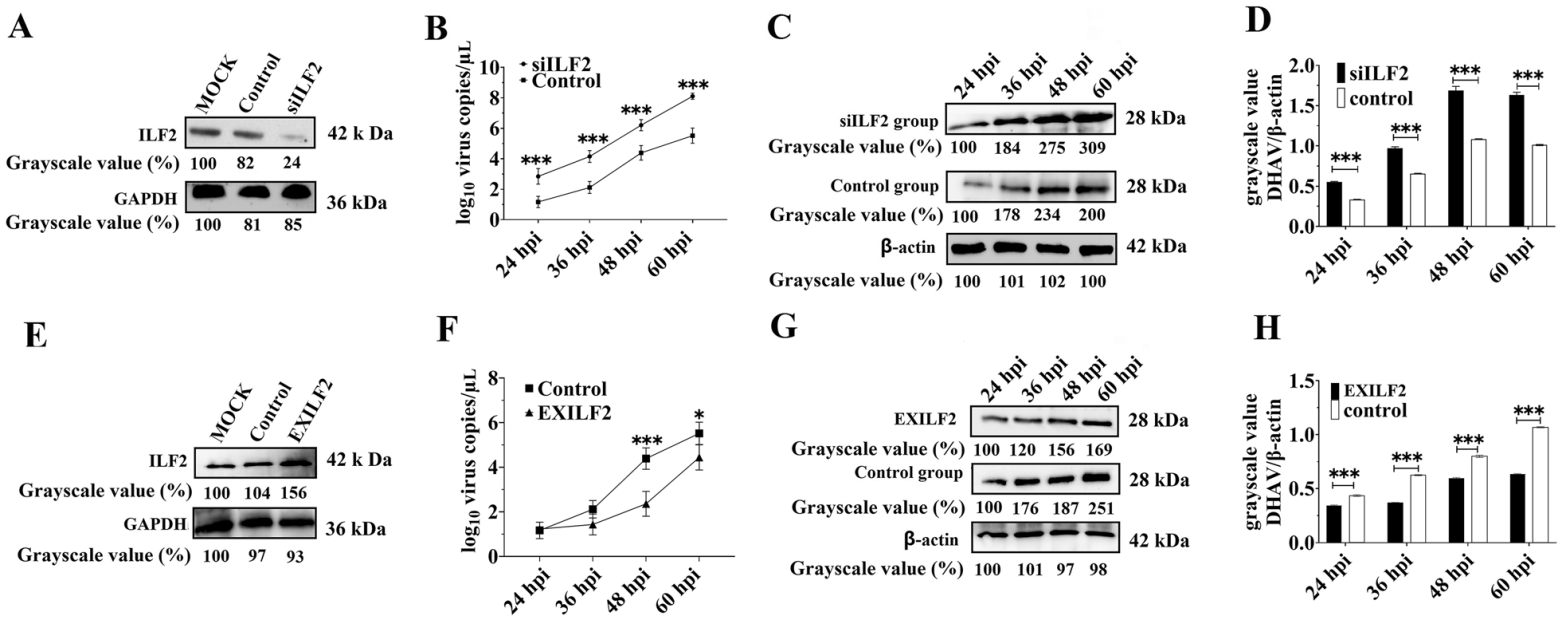
**ILF2 negatively regulates DHAV-1 replication.** **A** The silencing effect of ILF2 in DEFs at 60 hpt were measured through Western blot using anti-ILF2 antiserum (1:100). The Mock group indicated blank DEFs, the Control group indicated DEFs transfecting with negative control siRNA, and the siILF2 group indicated DEFs transfecting with siILF2. **B** qRT-PCR results of viral copies of DHAV-1 in control group and siILF2 group at 24, 36, 48, and 60 hpi. **C** Western blot analysis of the viral protein level of DHAV-1 in the siILF2 group and control group at 24, 36, 48, and 60 hpi. The anti-DHAV-1 antiserum (1:100) was used in this assay, and the gray scale value of each band was indicated **D**. **E** The expression levels of ILF2 in control group and EXILF2 group were detected by Western blot using anti-ILF2 antiserum (1:100) 48 h later. The Mock group indicated blank DEFs, the Control group indicated DEFs transfecting with pcDNA3.1/V5-His B vector, and the EXILF2 group indicated DEFs transfecting with pCDNA3.1-ILF2. **F** qRT-PCR results of viral copies of DHAV-1 in control group and EXILF2 group at 24, 36, 48, and 60 hpi. **G** The viral protein levels of DHAV-1 in the EXILF2 group and control group at 24, 36, 48, and 60 hpi were measured through Western blot using anti-DHAV-1 antiserum (1:100). **H** The grayscale value of each protein band in both groups was measured using ImageJ software. The y-axis represents the ratio of the gray value of the viral proteins to β-actin, while the x-axis represents the hours post-infection. * P < 0.05, ** P < 0.01, *** P < 0.001. Bars show the mean ± SD of three independent experiments (*n* = 3).

To identify the specific role of ILF2 during DHAV-1 replication, the expression of ILF2 was downregulated in DEFs using RNAi, and the silencing effect was measured using Western blot. At 60 hpt, the expression of ILF2 was highly reduced in the siRNA transfection group (named siILF2) compared with the control group (Figure 4A). Next, DEFs in the siILF2 and control groups were inoculated with approximately 10^4^ copies of DHAV-1. Viral growth characteristics in both groups were measured using qRT-PCR and Western blot. The qRT-PCR results showed that the viral copies in the siILF2 group increased 2.43-, 1.96-, 1.41-, and 1.47-fold between 24 and 60 hpt compared with the control group, while a 1.64-, 1.48-, 1.55-, and 1.61-fold increase of the grayscale value was observed in the siILF2 group compared with the control group (Figures 4B–D).

Next, the recombinant plasmids pCDNA3.1-ILF2 were transfected into DEFs (named EXILF2) to upregulate ILF2 expression. Equal copies of the pCDNA3.1 vectors were used as the control group. At 48 hpt, the Western blot results showed that ILF2 expression was highly increased in the EXILF2 group (Figure 4E). Subsequently, approximately 10^4^ copies of DHAV-1 were used to infect DEFs in the control and EXILF2 groups, and the viral load in both groups was compared. Both Western blot and qRT-PCR results showed that overexpression of ILF2 resulted in a sharp decrease in virus yield at each detection point. The number of viral copies in the EXILF2 group decreased by 5.28%, 32.29%, 46.22%, and 19.5% at 24 to 60 hpi compared with that in the control group. In comparison, a significant decrease of 21.51%, 41.01%, 25.13%, and 40.50% of the grayscale values was observed in the EXILF2 group at 24 to 60 hpi compared with that in the control group (Figures 4F–H). These results indicate that ILF2 negatively affects the replication of DHAV-1.

### ILF2 suppressed DHAV-1 replication through disrupting the RdRP activity of 3D^pol^

**Figure 5:**
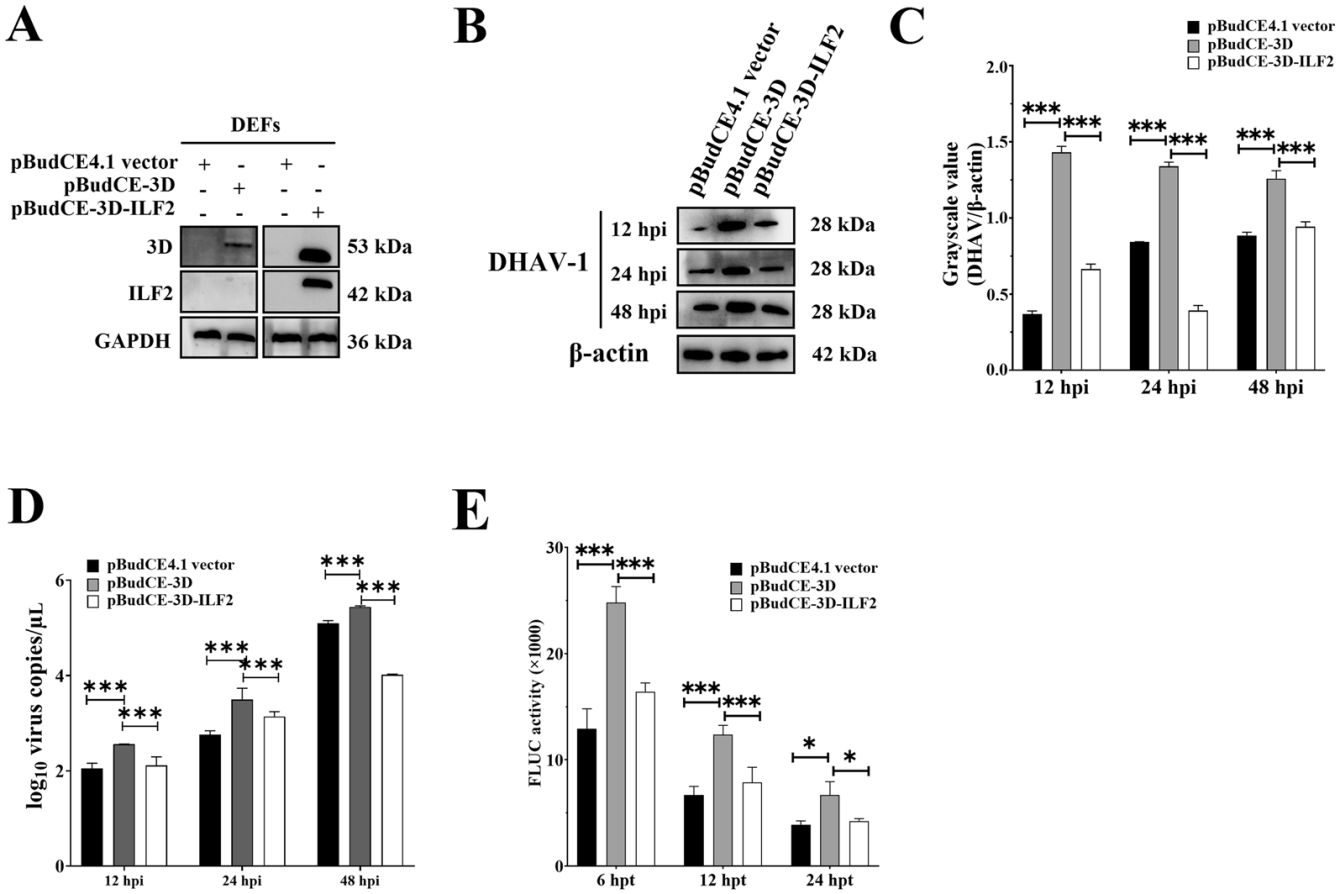
**ILF2 suppresses DHAV-1 replication by disrupting the RdRP activity of 3D**^**pol**^**.** **A** Western blot results of the expression level of 3D and ILF2 in DEFs transfected with pBudCE-3D or pBudCE-3D-ILF2 using an anti-MYC (1:3000) or anti-ILF2 (1:100) antibody. **B** Western blot analysis of DHAV-1 viral replication levels in DEFs transfected with the pBudCE4.1 vector, pBudCE-3D, or pBudCE-3D-ILF2 using anti-DHAV-1 antiserum (1:100); the grayscale value of each band **C**. **D** The growth characteristics of DHAV-1 in DEFs transfected with the pBudCE4.1 vector, pBudCE-3D, or pBudCE-3D-ILF2 measured using qRT-PCR. **E** Comparison of FLUC activities in DEFs transfected with the pBudCE4.1 vector, pBudCE-3D, or pBudCE-3D-ILF2. * P < 0.05, ** P < 0.01, *** P < 0.001. Bars show the mean ± SD of three independent experiments (*n* = 3).

It is apparent that the antiviral effect of ILF2 occurs through its binding to 3′-UTR of DHAV-1; however, ILF2 also binds 3D^pol^. Based on the aforementioned results, indicating that both ILF2 and 3D^pol^ interact with the 3′-UTR of DHAV-1, we hypothesized that ILF2 might suppress DHAV-1 replication by disrupting the RdRP activity of 3D^pol^. To confirm this hypothesis, DEFs were separately transfected with pBudCE4.1 vector, pBudCE-3D, or pBudCE-3D-ILF2 (Figure 5A). At 48 hpt, the DEFs in the three groups were inoculated with approximately 10^4^ copies of DHAV-1, and the numbers of viral copies in the three groups were compared through Western blotting at 12, 24, and 48 hpi. The results showed that the number of viral copies in the pBudCE-3D-ILF2-transfected group was highly suppressed compared to that in the pBudCE-3D-transfected group (Figures 5B–D), indicating that ILF2 disrupts 3D^pol^-mediated promotion of viral proliferation.

Next, we investigated whether ILF2 inhibits DHAV-1 replication by interfering with RdRP-mediated viral RNA translation efficiency. DEFs were separately transfected with the pBudCE4.1 vector, pBudCE-3D, or pBudCE-3D-ILF2. At 48 hpt, the DEFs in the three groups were then transfected with the translation reporter system 5′-UTR_FLUC_3′-UTR, and the FLUC activity was measured at 6, 12, and 24 hpt. FLUC activity in the pBudCE-3D group was highly higher compared to that in the pBudCE4.1 vector group, whereas FLUC activity in the pBudCE-3D-ILF2 group was highly suppressed compared to that in the pBudCE-3D group (Figure 5E), indicating that ILF2 suppressed DHAV-1 replication by decreasing the RdRP-mediated promotion of viral RNA translation efficiency.

### DHAV-1 infection suppresses ILF2 expression in vitro

**Figure 6:**
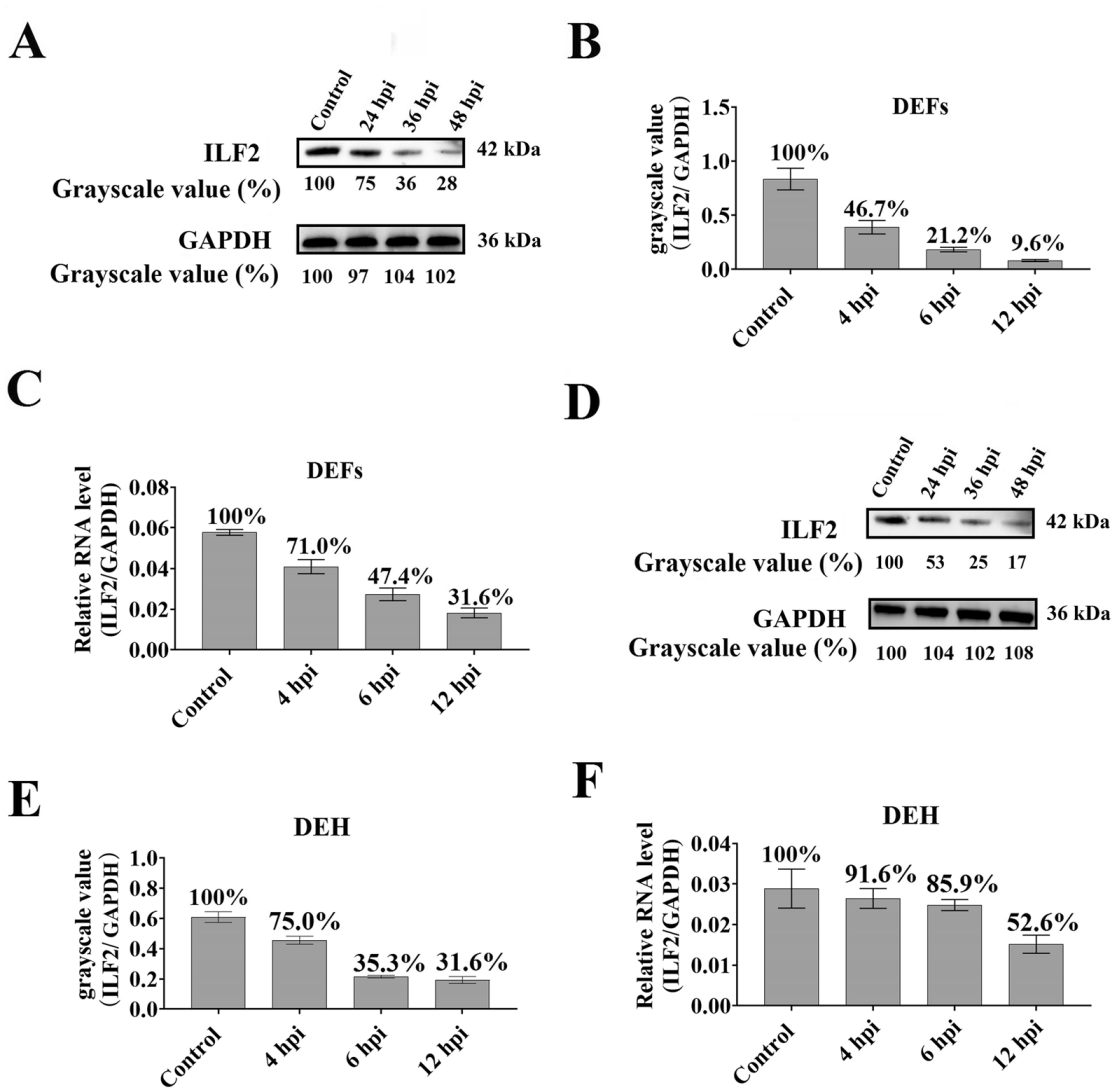
**Suppression of the expression of ILF2 by the replication of DHAV-1 in vitro.** **A** The expression levels of ILF2 in DEFs at 4, 6, and 12 hpi were measured through Western blot analysis using anti-ILF2 antiserum (1:100). **B** The grayscale value of each protein band was measured using ImageJ software. The y-axis represents the ratio of the ILF2 grayscale value to that of GAPDH, the x-axis represents the time post-infection (hours). **C** The qRT-PCR results show the expression level of ILF2 at 4, 6, and 12 hpi in DEFs. **D** Western blot analysis of ILF2 expression in DEH at 4, 6, and 12 hpi. The anti-ILF2 antiserum (1:100) was used. **E** The grayscale value of ILF2 in the DEH at different times post-infection. **F** The qRT-PCR results showed the expression level of ILF2 at 4, 6, and 12 hpi in DEH. Bars show the mean ± SD of three independent experiments (*n* = 3).

The changes a cellular protein undergoes upon viral infection provide a crucial indicator of the protein’s biological function. ILF2 was found to be differently expressed after DHAV infection [32]; however, the specific change trend remains unknown. Therefore, DEFs were infected with approximately 10^4^ copies of DHAV-1 to investigate the effect of DHAV-1 infection on ILF2 expression. The cell lysates were collected at 4, 6, and 12 hpi to perform qRT-PCR or Western blot analysis. Compared with that in the control group, the expression of ILF2 sharply decreased at all test points (Figures 6A, B) according to Western blot result; the qRT-PCR analysis also showed that the expression of ILF2 was strongly suppressed (Figure 6C). In addition, the variation in ILF2 expression in the DEHs was also measured using qRT-PCR and Western blot under the same conditions. The results showed that the expression of ILF2 was strongly suppressed after DHAV-1 infection (Figures 6D–F).

### DHAV-1 infection suppresses ILF2 expression in vivo

**Figure 7:**
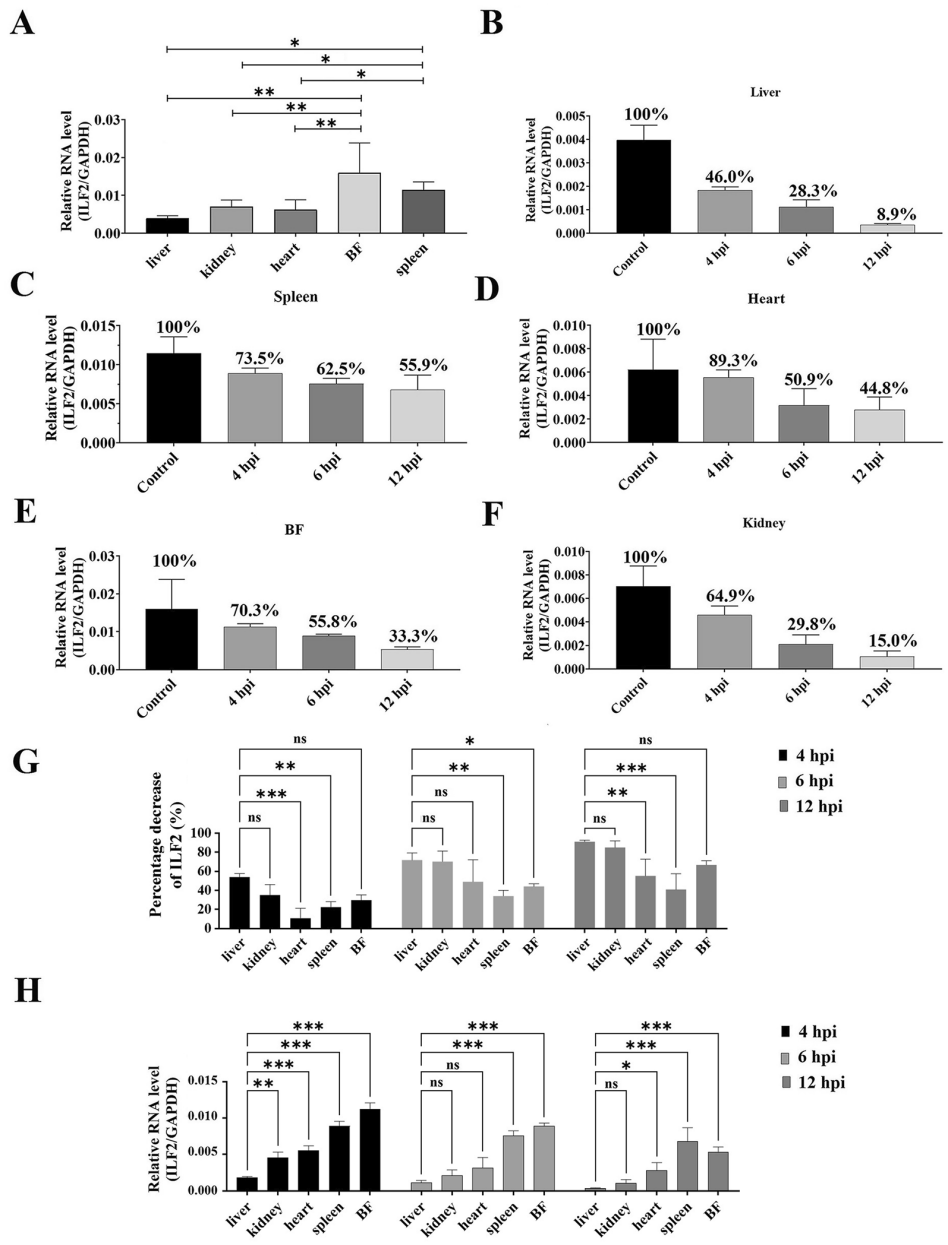
**Suppression of ILF2 expression by the replication of DHAV-1 in vivo.** **A** Expression levels of ILF2 in healthy ducklings’ liver, kidney, heart, spleen, and BF. **B–F** The expression levels of ILF2 in various tissues were measured through qRT-PCR at 4, 6, and 12 hpi. **G** Percentage decrease of ILF2 expression in each organ. **H** The transcription level of ILF2 in different tissues at different times post-infection. * P < 0.05, ** P < 0.01, *** P < 0.001. Bars show the mean ± SD of three independent experiments (*n* = 3).

We first measured the tissue distribution of ILF2 in 1-day-old ducklings using qRT-PCR. The highest expression of ILF2 was observed in the BF, followed by the spleen, kidney, heart, and liver (Figure 7A). Notably, the expression of ILF2 was higher in immune organs, including the BF and spleen, than in other tissues (Figure 7A). Next, the impact of DHAV-1 infection on ILF2 expression was investigated. The 1-day-old ducklings were inoculated intramuscularly with approximately 10^4^ copies of DHAV-1 particles. The ducklings in the control group were inoculated with the same amount of PBS, and the expression of ILF2 in different tissues was measured at 4, 6, and 12 hpi. Compared with the control group, ILF2 expression in all tissues decreased sharply after DHAV-1 infection (Figures 7B–F). The decrease in ILF2 expression varied greatly in the different organs: the liver showed the highest decrease at all measurement points, whereas the heart (at 4 hpi) and the spleen (at 6 and 12 hpi) showed the lowest decrease (Figure 7G). In addition, the liver showed the lowest ILF2 expression levels throughout the infection process (Figure 7H). In contrast, the expression of ILF2 in immune organs, including the BF and spleen, was highly higher compared with that in other tissues during the whole infection process (Figure 7H).

### Expression of ILF2 correlates with the proliferation of DHAV-1 in both DEFs and DEH

**Figure 8:**
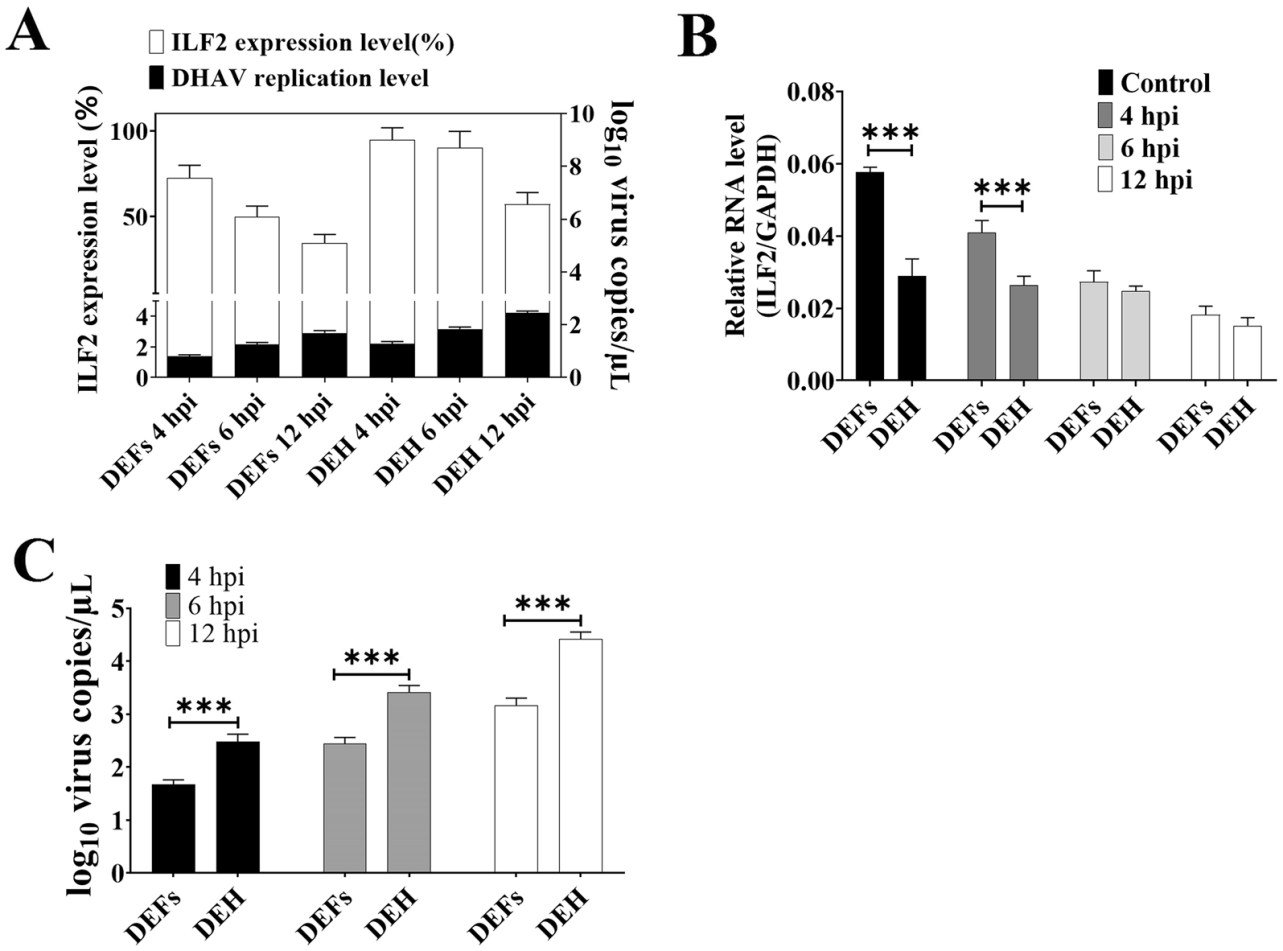
**Influence of ILF2 in the DHAV-1 viral load in vitro.** **A** Relationship between the expression of ILF2 and DHAV-1 viral load in DEFs and DEH. The left y-axis represents the logarithm of DHAV copies, and the right y-axis represents the average expression level of ILF2 (%). **B** Comparison between the relative RNA level of ILF2 in the DEFs and DEH groups. **C** Viral load of DHAV-1 in the DEFs and DEH groups were compared. *** P < 0.001. Bars show the mean ± SD of three independent experiments (*n* = 3).

Because both the tissue distribution of ILF2 and the viral load of DHAV-1 vary widely in different organs, we investigated whether the expression of ILF2 correlated with viral load in vitro. DEFs and DEH were infected with approximately 10^4^ viral particles, and the cell lysates were collected at different times post-infection for measurement using qRT-PCR. The results showed that the expression of ILF2 was negatively correlated with viral load (Figure 8A). In addition, the expression of ILF2 was consistently higher in DEFs than in DEH (Figure 8B). In contrast, the viral load was highly lower in DEFs than in DEH at all-time points tested (Figure 8C), indicating that the expression of ILF2 was negatively correlated with viral load in vitro.

### Expression of ILF2 correlates with the proliferation of DHAV-1 in vivo

**Figure 9:**
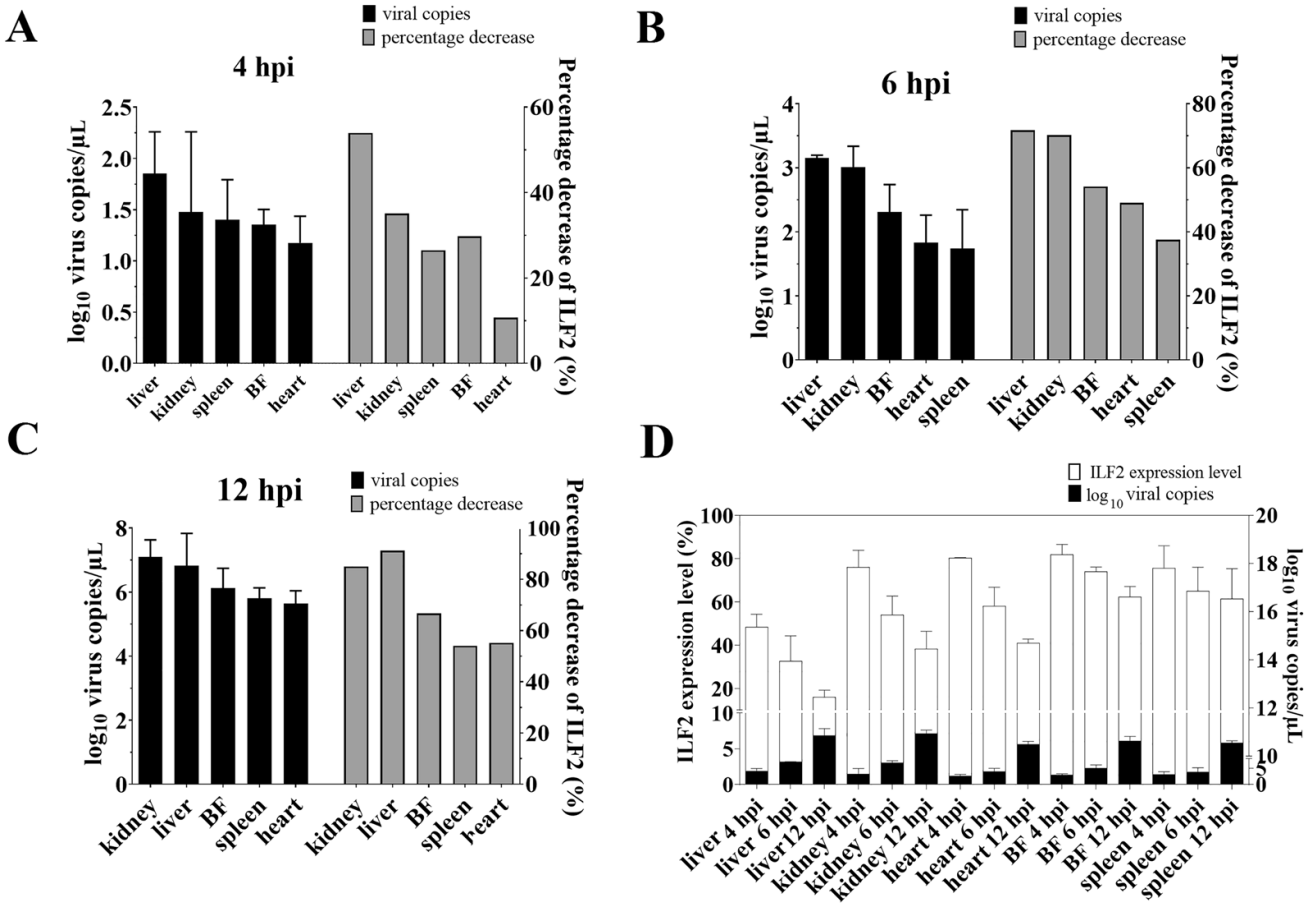
**Influence of ILF2 in the DHAV-1 viral load in vivo.** **A–C** Relationship between the percentage decrease in ILF2 expression and DHAV-1 viral load in various tissues at each detection point. The x-axis represents the different tissues, the left y-axis represents the viral load of DHAV-1, and the right y-axis represents the average percentage decrease in ILF2 expression (%). **D** Relationship between ILF2 expression and DHAV-1 viral load in various tissues at each detection point. The right y-axis represents the logarithm of DHAV-1 copies, and the left y-axis represents the average expression level of ILF2 (%). Bars show the mean ± SD of three independent experiments (*n* = 3).

Finally, we investigated whether the expression of ILF2 was correlated with viral load in different tissues of young ducklings after DHAV-1 infection, i.e., whether ILF2 affected viral tissue tropism. 1-day-old ducklings were injected intramuscularly with approximately 10^4^ copies of DHAV-1 particles. The viral load in the liver, kidney, spleen, heart, and BF was measured at 4, 6, and 12 hpi, and the relationship between viral load and the expression of ILF2 was compared. Notably, there was a positive correlation between the expression of ILF2 and DHAV-1 replication in various tissues at all detection time points (Figures 9A–C). In addition, the histogram results showed that the transcription level of ILF2 was inversely proportional to the proliferation level of DHAV-1 in all tissues examined (Figure 9D).

## Discussion

The infection cycles of DHAV-1 in host cells are a complex process involving a series of protein–RNA and protein–protein interactions. The interaction between virus and host factors is a key determinant of viral translation, viral genome replication, and post-translational modifications, aiding in our understanding of the molecular mechanism of viral pathogenesis and the development of antiviral drugs. Some cellular proteins may interact with viral RNA or proteins and affect replication cycles, while viral replication in the host cell could inversely alter the expression of cellular proteins. Currently, little is known about whether these host proteins interact with viral genome structures or viral proteins of DHAV-1, particularly with the host replication machinery, to regulate viral infection. In this study, we showed that ILF2 specifically interacted with the 3ʹ—UTR of DHAV-1 through an in vitro RNA pull-down assay (Figure 1). ILF2 and ILF3 are distinct proteins that shared a common domain known as domain associated with zinc fingers. ILF2 and ILF3 could dimerize through this common domain and affect viral replication by interacting with viral RNA [33]. However, the details of the interaction between the ILF2/ILF3 complex and viral RNA, such as what motifs are recognized by the complex and what the molecular consequences are of the complex association with viral RNA, are not yet well understood [33].

Our results also showed that ILF2 negatively regulated DHAV-1 replication (Figure 4), suggesting that ILF2 may act as a restriction factor for DHAV-1 replication by interfering with viral genome replication or viral protein expression. This is consistent with previous studies showing that silencing of ILF2 facilitates replication of IBDV [22], PRRSV [28], stomatitis virus [20], and influenza virus [24]. Interestingly, it has also been shown that ILF2 can specifically interact with RdRp, such as the viral VP1 of IBDV [22] and nsp9 of PRRSV [28], to prevent viral replication. The 3D^pol^ of DHAV-1, which also binds to the 3′-UTR to exert RdRP activity, is an important determinant of viral replication [9]. In the present study, we showed that ILF2 interacted with the 3D^pol^ of DHAV-1 both in vitro and in vivo (Figure 2). Because both 3D^pol^ and ILF2 specifically bound to the viral 3ʹ-UTR, we hypothesize that 3D^pol^ and ILF2 might competitively bond to the 3ʹ-UTR, and the competitive combination of ILF2 and 3D^pol^ might prevent 3D^pol^ from exerting the biological activity of RdRp (Figure 3), leading to a decrease in DHAV replication (Figure 5). Moreover, ILF2 might regulate viral replication through mediating the immune system. For example, ILF2 interacts with NOD-like receptor thermal protein domain associated protein 3 (NLRP3) and inhibits the activation of the NLRP3 inflammasome [34]. In the present study, we showed that ILF2 sharply decreased following DHAV-1 infection (Figures 6, 7). We hypothesize that the decrease in ILF2 expression might alleviate its inhibition of NLRP3 activation, resulting in high production of the downstream product of the NLRP3 signaling pathway, such as IL-1β, to negatively regulate DHAV-1 replication.

At last, we investigated the relationship between the DHAV-1 proliferation level and ILF2 expression level. Firstly, we noticed that the replication of DHAV strongly suppressed the expression of ILF2 (Figures 6, 7). Therefore, we hypothesized that DHAV-1 might facilitate viral replication by downregulating the expression of ILF2 based on the conclusion that ILF2 negatively regulated the replication of DHAV-1 (Figure 4). The cytopathic nature of DHAV-1 infection might directly suppress ILF2 expression, while the decrease of ILF2 might also due to the apoptosis or cell death. In addition, some microRNA, such as MicroRNA-7 (miR-7), might contribute to the decrease of ILF2 [35, 36]. Secondly, we speculated that the organs of healthy ducklings with low levels of ILF2 might be preferentially infected with DHAV-1 since ILF2 inhibits DHAV-1 replication. Previous studies have shown that DHAV-1 preferentially infects liver tissue since the viral genome could only be detected in liver as early as 1 hpi [37]. Therefore, the highly low initial transcription levels of ILF2 in liver tissue (Figure 7A) might contribute to the hepatotropism of DHAV-1. Thirdly, we showed a correlation between the expression of ILF2 and viral load in primary cells (Figure 8). The expression of ILF2 was higher in the DEF group than in the DEH group at all-time points (Figure 8D). The opposite was observed with the viral load in the DEF group, which was consistently lower than that in the DEH group (Figure 8E), suggesting that the higher expression of ILF2 may lead to more effective inhibition of DHAV-1 replication in vitro. Finally, the expression of ILF2 might affect the viral load of DHAV-1 in different tissues of ducklings (Figure 9). For example, the expression of ILF2 was highly higher in immune organs, such as the spleen and BF, than in the liver (Figure 7A), whereas the viral load was lower in immune organs than in the liver tissue [31], indicated that the higher expression of ILF2 in immune organs was more effective in preventing viral replication. Moreover, liver tissue, which had the lowest expression of ILF2 at all test time points (Figure 7H), showed the highest viral load throughout the infection process [37], suggesting that DHAV-1 may replicate more effectively in organs with lower ILF2 expression. To sum up, the viral load in primary cells and various tissues of young ducklings was negatively correlated with ILF2 expression (Figures 8, 9), suggesting that ILF2 may influence the viral tissue tropism of DHAV-1.

In conclusion, ILF2 suppressed viral proliferation of DHAV-1 by disrupting the RdRP-mediated promotion of viral RNA translation efficiency. In addition, viral replication strongly inhibits the expression of ILF2, which is beneficial for virus reproduction in host cells. At last, the ILF2 expression level negatively correlated with the viral proliferation level in various organs, indicating a possible role of ILF2 on regulating tissue tropism.

## Data Availability

The datasets analyzed in this study are available from the corresponding author upon reasonable request.

## References

[1] Levine P, Fabricant J (1950). A hitherto-undescribed virus disease of ducks in North-America. Cornell Vet.

[2] Haider SA, Calnek BW (1979). In vitro isolation, propagation, and characterization of duck hepatitis virus type III. Avian Dis.

[3] Toth TE (1969). Studies of an agent causing mortality among ducklings immune to duck virus hepatitis. Avian Dis.

[4] Wang L, Pan M, Fu Y, Zhang D (2008). Classification of duck hepatitis virus into three genotypes based on molecular evolutionary analysis. Virus Genes.

[5] Knowles N, Hovi T, Hyypiä T, King A, Lindberg A, Pallansch M, Palmenberg A, Simmonds P, Skern T, Stanway G, King AMQ, Adams MJ, Carstens EB (2012). Picornaviridae. Virus taxonomy: classification and nomenclature of viruses: Ninth report of the international committee on taxonomy of viruses.

[6] Kim MC, Kwon YK, Joh SJ, Lindberg AM, Kwon JH, Kim JH, Kim SJ (2006). Molecular analysis of duck hepatitis virus type 1 reveals a novel lineage close to the genus *Parechovirus* in the family *Picornaviridae*. J Gen Virol.

[7] Tseng CH, Knowles NJ, Tsai HJ (2007). Molecular analysis of duck hepatitis virus type 1 indicates that it should be assigned to a new genus. Virus Res.

[8] Kim MC, Kwon YK, Joh SJ, Kim SJ, Tolf C, Kim JH, Sung HW, Lindberg AM, Kwon JH (2007). Recent Korean isolates of duck hepatitis virus reveal the presence of a new geno—and serotype when compared to duck hepatitis virus type 1 type strains. Arch Virol.

[9] Chen JH, Zhang RH, Lin SL, Li PF, Lan JJ, Song SS, Gao JM, Wang Y, Xie ZJ, Li FC, Jiang SJ (2018). The functional role of the 3' untranslated region and poly(A) tail of duck hepatitis A virus type 1 in viral replication and regulation of IRES-mediated translation. Front Microbiol.

[10] Zhang Y, Cao Q, Wang M, Jia R, Chen S, Zhu D, Liu M, Sun K, Yang Q, Wu Y, Zhao X, Chen X, Cheng A (2017). The 3D protein of duck hepatitis A virus type 1 binds to a viral genomic 3' UTR and shows RNA-dependent RNA polymerase activity. Virus Genes.

[11] Butcher SJ, Grimes JM, Makeyev EV, Bamford DH, Stuart DI (2001). A mechanism for initiating RNA-dependent RNA polymerization. Nature.

[12] Min BS, Han KR, Lee JI, Yang JM (2012). cDNA cloning of Korean human norovirus and nucleotidylylation of VPg by norovirus RNA-dependent RNA polymerase. J Microbiol.

[13] Guan D, Altan-Bonnet N, Parrott AM, Arrigo CJ, Li Q, Khaleduzzaman M, Li H, Lee CG, Pe'ery T, Mathews MB (2008). Nuclear factor 45 (NF45) is a regulatory subunit of complexes with NF90/110 involved in mitotic control. Mol Cell Biol.

[14] Shi C, Yang Y, Yu J, Meng F, Zhang T, Gao Y (2017). The long noncoding RNA LINC00473, a target of microRNA 34a, promotes tumorigenesis by inhibiting ILF2 degradation in cervical cancer. Am J Cancer Res.

[15] Shamanna RA, Hoque M, Lewis-Antes A, Azzam EI, Lagunoff D, Pe'ery T, Mathews MB (2011). The NF90/NF45 complex participates in DNA break repair via nonhomologous end joining. Mol Cell Biol.

[16] Kiesler P, Haynes PA, Shi L, Kao PN, Wysocki VH, Vercelli D (2010). NF45 and NF90 regulate HS4-dependent interleukin-13 transcription in T cells. J Biol Chem.

[17] Sakamoto S, Aoki K, Higuchi T, Todaka H, Morisawa K, Tamaki N, Hatano E, Fukushima A, Taniguchi T, Agata Y (2009). The NF90-NF45 complex functions as a negative regulator in the microRNA processing pathway. Mol Cell Biol.

[18] Todaka H, Higuchi T, Yagyu K, Sugiyama Y, Yamaguchi F, Morisawa K, Ono M, Fukushima A, Tsuda M, Taniguchi T, Sakamoto S (2015). Overexpression of NF90-NF45 represses myogenic microRNA biogenesis, resulting in development of skeletal muscle atrophy and centronuclear muscle fibers. Mol Cell Biol.

[19] Kuwano Y, Pullmann R, Marasa BS, Abdelmohsen K, Lee EK, Yang X, Martindale JL, Zhan M, Gorospe M (2010). NF90 selectively represses the translation of target mRNAs bearing an AU-rich signature motif. Nucleic Acids Res.

[20] Pfeifer I, Elsby R, Fernandez M, Faria PA, Nussenzveig DR, Lossos IS, Fontoura BM, Martin WD, Barber GN (2008). NFAR-1 and -2 modulate translation and are required for efficient host defense. Proc Natl Acad Sci U S A.

[21] Adriani O, Barbarino GC, Bazilevskaya GA, Bellotti R, Boezio M, Bogomolov EA, Bonechi L, Bongi M, Bonvicini V, Bottai S, Bruno A, Cafagna F, Campana D, Carlson P, Casolino M, Castellini G, De Pascale MP, De Rosa G, Fedele D, Galper AM, Grishantseva L, Hofverberg P, Leonov A, Koldashov SV, Krutkov SY, Kvashnin AN, Malvezzi V, Marcelli L, Menn W, Mikhailov VV (2009). New measurement of the antiproton-to-proton flux ratio up to 100 GeV in the cosmic radiation. Phys Rev Lett.

[22] Stricker RL, Behrens SE, Mundt E (2010). Nuclear factor NF45 interacts with viral proteins of infectious bursal disease virus and inhibits viral replication. J Virol.

[23] Acar N, Bonhomme B, Joffre C, Bron AM, Creuzot-Garcher C, Bretillon L, Doly M, Chardigny JM (2006). The retina is more susceptible than the brain and the liver to the incorporation of trans isomers of DHA in rats consuming trans isomers of alpha-linolenic acid. Reprod Nutr Dev.

[24] Wang P, Song W, Mok BW, Zhao P, Qin K, Lai A, Smith GJ, Zhang J, Lin T, Guan Y, Chen H (2009). Nuclear factor 90 negatively regulates influenza virus replication by interacting with viral nucleoprotein. J Virol.

[25] Gomila RC, Martin GW, Gehrke L (2011). NF90 binds the dengue virus RNA 3' terminus and is a positive regulator of dengue virus replication. PLoS ONE.

[26] Li Y, Belshan M (2016). NF45 and NF90 bind HIV-1 RNA and modulate HIV gene expression. Viruses.

[27] Murphy J, Hall WW, Ratner L, Sheehy N (2016). Novel interactions between the HTLV antisense proteins HBZ and APH-2 and the NFAR protein family: implications for the HTLV lifecycles. Virology.

[28] Wen X, Bian T, Zhang Z, Zhou L, Ge X, Han J, Guo X, Yang H, Yu K (2017). Interleukin-2 enhancer binding factor 2 interacts with the nsp9 or nsp2 of porcine reproductive and respiratory syndrome virus and exerts negatively regulatory effect on the viral replication. Virol J.

[29] Cui X, Qian P, Rao T, Wei Y, Zhao F, Zhang H, Chen H, Li X (2019). Cellular interleukin enhancer-binding factor 2, ILF2, inhibits Japanese encephalitis virus replication in vitro. Viruses.

[30] Chen J, Zhang R, Lan J, Lin S, Li P, Gao J, Wang Y, Xie ZJ, Li FC, Jiang SJ (2019). IGF2BP1 significantly enhances translation efficiency of duck hepatitis A virus type 1 without affecting viral replication. Biomolecules.

[31] Lin SL, Cong RC, Zhang RH, Chen JH, Xia LL, Xie ZJ, Wang Y, Zhu YL, Jiang SJ (2016). Circulation and in vivo distribution of duck hepatitis A virus types 1 and 3 in infected ducklings. Arch Virol.

[32] Wu F, Lu F, Fan X, Chao J, Liu C, Pan Q, Sun H, Zhang X (2020). Immune-related miRNA-mRNA regulation network in the livers of DHAV-3-infected ducklings. BMC Genomics.

[33] Wolkowicz UM, Cook AG (2012). NF45 dimerizes with NF90, Zfr and SPNR via a conserved domain that has a nucleotidyltransferase fold. Nucleic Acids Res.

[34] Jin J, Li A, Wang W, Wu J (2018). Interleukin-enhanced binding factor 2 interacts with NLRP3 to inhibit the NLRP3 inflammasome activation. Biochem Biophys Res Commun.

[35] Bi Y, Shen W, Min M, Liu Y (2017). MicroRNA-7 functions as a tumor-suppressor gene by regulating ILF2 in pancreatic carcinoma. Int J Mol Med.

[36] Huang Y, Wang W, Xu Z, Pan J, Zhao Z, Ren Q (2018). *Eriocheir sinensis* microRNA-7 targets crab *Myd88* to enhance white spot syndrome virus replication. Fish Shellfish Immunol.

[37] Chen J, Zhang R, Lin S, Li P, Lan J, Xie Z, Wang Y, Jiang S (2017). Construction and characterization of an improved DNA-launched infectious clone of duck hepatitis a virus type 1. Virol J.

